# Why diagnostic and nuclear medical physicists matter in academic medical centers: A perspective from a radiology department chair and a medical physicist

**DOI:** 10.1002/acm2.70255

**Published:** 2025-09-01

**Authors:** Jie Zhang, M. Elizabeth Oates

**Affiliations:** ^1^ Department of Radiology University of Kentucky College of Medicine Lexington Kentucky USA

Diagnostic and nuclear medical physicists are central to quality, safety, and innovation in radiology, yet their contributions are often underrecognized. Since 2011, our department has developed a Division of Diagnostic & Nuclear Medical Physics from the ground up. The team now includes three ABR‑certified PhD diagnostic physicists, a MS health physicist, a part‑time PhD MRI scientist, a physicist assistant, and two residents. From time to time, we are asked who physicists are, what they do, and why radiology needs an integrated physics division. As a department chair (MEO) and a physicist (JZ), we offer a joint perspective on why physicists are essential and how departments can better leverage and empower them as faculty, leaders, and innovators.

## 1 WHO ARE PHYSICISTS?

The American Association of Physicists in Medicine (AAPM) defines medical physics as a branch of applied physics concerned with the application of the concepts and methods of physics to the diagnosis and treatment of human disease. Clinical medical physicists specialize in diagnostic imaging, nuclear medicine, or therapy. Their shared responsibility is to ensure that radiation is used safely and effectively to achieve clinical goals. In theory, medical physicists, regardless of specialty, play an equally complementary role to other medical professionals. However, in reality, diagnostic and nuclear physicists often must justify their roles, while therapeutic physicists are widely accepted because their work is visibly tied to treatment planning, delivery, documentation, and reimbursement. Two factors drive the disparity for diagnostic and nuclear physics: the lack of reimbursement mechanisms and inconsistent perceptions of physicist roles in imaging. Although the AAPM, the American College of Radiology (ACR), and the literature outline these responsibilities, many radiology teams still consider diagnostic physicists as optional.^1^, ^2^, ^3^, ^4^ Samei and Seibert observed that even substantial academic centers undervalue this potential, with groups either nonexistent or understaffed and poorly integrated into patient care^5^. Since the time of Roentgen, medical physicists have bridged the gap between physics and medicine and catalyzed translational advances to improve patient care. Today, as imaging grows more complex, data‐intensive, and technology‐driven, physicists are indispensable in ensuring quality, safety, and clinical excellence.

## 2 CLINICAL IMPACT

Physicists add clinical value to radiology practice across imaging modalities. At our institution, physicists are embedded in daily operations, overseeing more than 200 imaging systems at 11 locations. They lead acceptance testing and calibration, integrate new technologies, and monitor performance. They optimize radiation dose by reviewing protocols and monitoring exposure to balance image quality and safety. Their work supports state and federal requirements, the Joint Commission, and ACR accreditation, including our designation as an ACR Diagnostic Imaging Center of Excellence (DICOE) since 2018. Physicists sustain this status through continuous monitoring, documentation, and process improvement. Physicists lead radiation protection for patients and staff, conduct incident reviews, and guide policy on shielding, contamination response, and training. They contribute directly to patient care through fetal dose assessments, peak skin dose calculations, and individualized radiation consultations.

In radiotheranostics, physicists support treatment planning, activity calculation, and patient‑specific dosimetry. Their involvement has supported the growth of our Y‑90 microsphere program and radiopharmaceutical therapies. Steady physics leadership has been instrumental in securing the Society of Nuclear Medicine and Molecular Imaging (SNMMI) Comprehensive Radiotheranostics Center of Excellence designation in 2024. Working with radiation safety specialists, physicists maintain a patient‑facing role across targeted radiopharmaceutical therapies, including oral radioiodine and parenteral therapies for neuroendocrine tumor and prostate cancer.

Departments that invest in dedicated physics staffing, structured roles, and collaborative leadership frameworks realize returns through quality, efficiency, cost control, and risk reduction. Although physicists do not bill CPT codes, embedding them in clinical and operational decisions enables higher‑value care.

## 3 EDUCATIONAL IMPACT

The role of physicists as educators should be valued equivalently to clinical teaching. Their leadership in educational innovation strengthens board exam performance, cultivates curiosity, and prepares future clinicians for a rapidly evolving technological landscape.

Physics is foundational to radiology but underrepresented in clinical education. Our physicists have pioneered educational innovations to close this gap. They developed a week‑long rotation for diagnostic radiology residents, supported by a Radiological Society of North America (RSNA) Education Scholar Grant, that combines lectures and hands‑on labs and has served as a model for other programs.^6^ To support longitudinal learning, we implemented recurring initiatives such as “Artifact of the Month” and “Physics Question of the Week.” Residents submitted clinical artifacts with causes and mitigation strategies, which were reviewed and discussed periodically. Weekly, two physics questions are sent to residents with immediate feedback to reinforce core concepts. To address the growing role of Artificial Intelligence (AI) in medicine, our physicists created an 8‑h, hands‑on AI education course covering image processing, radiomics, and deep learning. Coding exercises and case studies link concepts to clinical imaging, and evaluations show increased resident confidence and understanding of AI in practice.

Our physicist team plays a central role in graduate medical physics training. They direct advanced graduate courses and manage practicums in CAMPEP‑accredited Master's and PhD programs. They teach two advanced graduate‐level medical physics courses and one research course, and manage the clinical practicum for students in the imaging track. With an AAPM/RSNA grant, we successfully launched Kentucky's first CAMPEP‑accredited Diagnostic Imaging Physics Residency Program, which now trains two residents per year.

Physicists provide targeted education for technologists, referring providers, and hospital staff on dose reduction and safety. In MRI, physicists train teams on ferromagnetic screening, implant and device compatibility, and high‑field hazards. These efforts support compliance and strengthen quality assurance across the enterprise.

## 4 SCHOLARLY CONTRIBUTIONS

Medical physicists pursue applied research that improves diagnostic accuracy, reduces dose, optimizes workflow, and solves safety problems. Recent examples include evaluating Virtual Grid technology in portable radiography to guide adoption decisions^7^ and investigating S‑distortion during fluoroscopically‐guided orthopedic procedures to identify sources and mitigation strategies.^8^ Our physicists’ research spans both foundational topics in imaging science and cutting‐edge topics such as AI and radiomics for clinical decision support. For example, we are developing deep learning methods for CT image quality assessment to optimize protocols and reduce exposure.^9^ Physicists serve as principal investigators and co‑investigators on internally and externally funded studies, including NIH and PCORI awards, and contribute to clinical trials such as ^177^Lu‑DOTATATE in lung cancer and the HERO Phase 1b ION269 study. Beyond projects, physicists mentor trainees and disseminate findings through publications and presentations.

## 5 PROFESSIONAL AND COMMUNITY SERVICE

Physicists enhance institutional and national leadership by shaping policy, advancing quality initiatives, and supporting accreditation. Nationally, our physicists hold leadership roles, including chairing the AAPM Diagnostic Radiology Resident Physics Curriculum Working Group that led a major curriculum update. Others serve on American Board of Radiology (ABR) Examination committees, ACR committees, and more than 20 panels across the AAPM, the American Board of Medical Physics (ABMP), the RSNA, and the Health Physics Society (HPS). These roles align departmental practice with national standards and raise institutional visibility. Physicists serve as editors and reviewers for journals and grant agencies, including the NIH, the RSNA, the Department of Defense, and internal programs, strengthening scholarship and funding. Locally, physicists lead capital and facility planning, protocol optimization, and safety across modalities, and serve on University (Radiation Safety, HealthCare Radiation Safety) and departmental (Executive Advisory, Education, Research) committees as well as multiple Clinical Practice and Quality Improvement teams.

## 6 STRATEGIC INTEGRATION OF PHYSICISTS INTO RADIOLOGY DEPARTMENTS

Many institutions employ physicists through the hospital with courtesy academic titles. Our department recognizes radiological medical physics as an independent division and embeds physicists as full academic faculty on par with clinical divisions. While faculty status typically requires a PhD, qualified MS‑trained physicists may serve as instructors. This model supports meaningful engagement across clinical, educational, and research missions. It affirms that physicists are strategic faculty partners. Table 1 summarizes this tripartite mission structure, outlining the responsibilities, justification mechanisms, and institutional benefits.

**TABLE 1:** Physicist roles by mission.

Mission	Responsibilities	Justification	Benefits
Clinical	Modality‐based duties — Technology assessment — Purchasing specifications — Acceptance testing — Clinical integration of new technology — Calibration — Safety surveys — Shielding plan development — Protocol optimization — Accreditation programs — Time‐based duties — Consultation with radiologists on patient dose and image quality — Consultation with physicians/patients on radiation safety — Planning for new imaging and therapy facilities — Oversight of radiation safety programs — Committee participation — Safety and technology committees	Based on modality count and AAPM Report 33 FTE guidelines Specific tasks such as acceptance testing and accreditation	Maximizes imaging investment Ensures compliance Bridges interdisciplinary communication Supports highest standards of care
Education	Didactic and lab‐based teaching for residents Hands‐on instruction for technologists and equipment users Continuing education for — Imaging faculty physicians — Residents and fellows — Nurses and technologists — Referring providers Institutional safety education — Topics such as pregnancy and medical radiation	Comparable to clinical teaching Based on lecture volume and breadth of instructional engagement	Improves board exam performance Builds technical fluency Enhances clinical decision‐making
Research	Conducting clinical research and supporting the development of new technologies and imaging procedures Mentoring of students, residents, and fellows Participating in academic output including peer‐reviewed publications, abstracts, presentations, and posters/exhibits	Same as other faculty members (e.g., radiologists) 10% protected time for research duties	Advances clinical technology Supports innovation Builds academic reputation

## 7 CHALLENGES

Despite their clinical and academic contributions, diagnostic and nuclear medical physicists often lack formal reimbursement pathways (not “providers”), and their roles are inconsistently defined across institutions. Many remain underrecognized or structurally isolated from the core faculty. Enduring barriers include limited leadership awareness of physicist scope; administrative structures that exclude physicists from strategic planning; weak metrics for impact on safety, quality, and efficiency; difficulty justifying employees without direct revenue; and recruitment and retention challenges due to unclear career paths and promotion criteria. The central challenge is to build a sustainable infrastructure and financial model that embeds physicists into the fabric of radiology practice.

## 8 PERSPECTIVE

Diagnostic imaging continues to evolve, bringing new opportunities and challenges. AI, radiotheranostics, and quantitative imaging are transforming practice, and diagnostic and nuclear medical physicists are uniquely positioned to lead this evolution. Their expertise moves departments from reactive troubleshooting to proactive quality and safety design. To truly realize the clinical, operational, and strategic value that diagnostic and nuclear medical physicists offer, radiology departments must move beyond ad hoc involvement and instead adopt intentional models of faculty integration, staffing investment, and leadership inclusion. This includes equitable faculty status, defined teaching roles, protected research time, and a seat at the strategic table. Just as no radiology department can function without radiologists, no forward‐looking department should function without diagnostic physicists. They are essential.

## CONFLICT OF INTEREST STATEMENT

The authors declare no conflicts of interest.
